# Efficacy and Safety of Cabotegravir–Rilpivirine in PLWH: A Real-World Study

**DOI:** 10.3390/v17111417

**Published:** 2025-10-24

**Authors:** Giuseppe Nicolò Conti, Serena Spampinato, Andrea De Vito, Andrea Marino, Teresa Cirelli, Viviana Coco, Alessia Mirabile, Rossella Fontana del Vecchio, Antonina Franco, Arturo Montineri, Chiara Fasca, Chiara Gullotta, Michele Salvatore Paternò Raddusa, Ylenia Russotto, Sonia Sofia, Grazia Pantò, Claudia Calì, Roberto Bruno, Eugenia Pistarà, Nunziatina Villari, Carmelo Iacobello, Bruno Cacopardo, Benedetto Maurizio Celesia, Giovanni F. Pellicanò, Francesco P. Antonucci, Sergio Lo Caputo, Giordano Madeddu, Giuseppe Nunnari, Emmanuele Venanzi Rullo

**Affiliations:** 1 Department of Biomedical and Biotechnological Sciences, University of Catania, 95123 Catania, Italy; giuseppecontichimica@gmail.com; 2 Unit of Infectious Diseases, ARNAS “Garibaldi Nesima” Hospital, Department of Clinical and Experimental Medicine, University of Catania, 95122 Catania, Italy; dott.ssaserenaspampinato@gmail.com (S.S.); andrea.marino@unict.it (A.M.); teresa.cirelli@gmail.com (T.C.); viviana.coco@gmail.com (V.C.); alessia.mirabile@gmail.com (A.M.); chiaragullotta96@gmail.com (C.G.); michelepat93@gmail.com (M.S.P.R.); roberto.bruno@unict.it (R.B.); eugeniapistara@gmail.com (E.P.); nancy.villari@gmail.com (N.V.); bruno.cacopardo@unict.it (B.C.); bmcelesia@gmail.com (B.M.C.); 3 Unit of Infectious Diseases, “G. Martino” University Hospital, Department of Clinical and Experimental Medicine, University of Messina, 98125 Messina, Italy; ylenia.russ@gmail.com (Y.R.); giovanni.pellicano@unime.it (G.F.P.); evenanzirullo@unime.it (E.V.R.); 4 Unit of Infectious Diseases, Department of Medicine, Surgery and Pharmacy, University of Sassari, 07100 Sassari, Italy; andreadevitoaho@gmail.com (A.D.V.); giordano@uniss.it (G.M.); 5 Unit of Infectious Diseases, Umberto I Hospital, ASP, 96100 Siracusa, Italy; r.fontanadelvecchio@gmail.com (R.F.d.V.); antoninafranco18@gmail.com (A.F.); 6 Unit of Infectious Diseases, “G. Rodolico-S. Marco” University Hospital, University of Catania, 95121 Catania, Italy; a.montineri@libero.it (A.M.); chiara.frasca@gmail.com (C.F.); 7 Unit of Infectious Diseases, AOE “Cannizzaro”, 95126 Catania, Italy; sonia.sofia@email.it (S.S.); graziapanto@tiscali.it (G.P.); carmelo.iacobello@gmail.com (C.I.); 8 Unit of Infectious Diseases, ASP Ragusa, 97100 Ragusa, Italy; claudia.cali@studenti.unime.it; 9 Unit of Infectious Diseases, Department of Medical and Surgical Sciences, University of Foggia, 71122 Foggia, Italy; antonuccifp@gmail.com (F.P.A.); sergio.locaputo@unifg.it (S.L.C.)

**Keywords:** long-acting antiretroviral therapy (LA-ART), cabotegravir–rilpivirine, virological suppression, real-world evidence, HIV treatment adherence

## Abstract

Background: Long-acting injectable antiretroviral therapy (LA-ART) with cabotegravir and rilpivirine (CAB + RPV) has emerged as a promising alternative to daily oral regimens for people living with HIV (PLWH), particularly those facing adherence challenges. While clinical trials have demonstrated its efficacy, real-world evidence remains limited. Methods: This retrospective, multicenter study evaluated the efficacy and safety of CAB + RPV in 160 virologically suppressed PLWH across eight Italian infectious disease units. Participants received intramuscular CAB (600 mg) and RPV (900 mg) every eight weeks without an oral lead-in phase. Clinical, immunological, and biochemical parameters were assessed at baseline and after 24 weeks. Results: At week 24, 96.25% of participants maintained virological suppression, and the proportion of individuals with target-not-detected viral load increased from 71% to 76%. Only one case of virological failure was observed. Significant immunological improvements included an increase in the CD4+/CD8+ ratio (*p* = 0.0038) and a reduction in CD8+ T-cell count (*p* = 0.0150). Biochemical analysis showed a decrease in serum creatinine (*p* < 0.0001) and an increase in HDL cholesterol (*p* = 0.0223). Treatment discontinuation occurred in 3.75% of participants, primarily due to adverse events or psychological factors. Conclusions: CAB + RPV demonstrated high efficacy and tolerability in a real-world setting, with favorable immunological and metabolic outcomes. These findings support its use as a viable therapeutic option for PLWH, especially those with adherence barriers. Further long-term studies are warranted to confirm these results across broader populations.

## 1. Introduction

Antiretroviral therapy (ART) has transformed the management of people living with HIV (PLWH), enabling sustained viral suppression, reducing morbidity and mortality, and improving health-related quality of life (HRQoL). Despite these advances, adherence to daily oral regimens remains a significant challenge, often hindered by pill burden, side effects, stigma, and psychosocial factors. Suboptimal adherence is associated with disease progression, increased healthcare costs, and diminished quality of life [1].

Long-acting (LA) injectable ART presents a promising alternative for individuals struggling with daily oral therapy. By reducing dosing frequency, LA formulations may enhance adherence and patient satisfaction. Surveys across diverse populations, including adolescents, women, and minority groups, consistently report a strong interest in LA-ART [2]. In South Africa, for instance, young PLWH cited medication stock-outs, stigma, and pill burden as reasons for preferring injectable options.

Clinical trials have demonstrated the efficacy and acceptability of LA-ART [3,4]. In Italy, a multicenter survey of 242 patients found that 90% were interested in switching to LA-ART, with a preference for both hospital-based and home-based administration [5]. Cross-national comparisons, such as between Spain and the U.S., reveal differing preferences regarding injection frequency and oral therapy [6].

Currently, three LA antiretroviral therapies are approved for HIV-1 treatment. The European Medicines Agency (EMA) has authorized VOCABRIA© (cabotegravir) and REKAMBYS© (rilpivirine) for co-administration, while the U.S. Food and Drug Administration (FDA) approved the co-packaged CABENUVA©. These therapies are administered monthly or bimonthly via intramuscular injection. Additional LA agents include lenacapavir (LEN), a capsid inhibitor [7], and Ibalizumab [8], a monoclonal antibody.

Adherence during pregnancy and the postpartum period is critical for maternal health and the prevention of vertical transmission. LA-ART may help overcome adherence barriers in this population, with emerging data supporting the safety of cabotegravir during pregnancy [9].

The CAB + RPV combination is indicated for maintenance therapy in adults with undetectable viral loads (<50 copies/mL) and no resistance to NNRTIs or INSTIs [10]. However, factors such as obesity [11] and gender-related adipose distribution [12] may affect drug pharmacokinetics, potentially requiring longer needles or adjusted dosing strategies.

Cabotegravir has a favorable drug interaction profile, is primarily metabolized by hepatic UGT1A1, and undergoes minimal renal excretion. Rilpivirine may affect renal transporters and creatinine levels, though genetic variants like Gilbert’s syndrome do not necessitate dose modifications.

Pharmacokinetic studies have shown prolonged drug exposure following intramuscular administration [10]. For example, the ECLAIR study detected cabotegravir levels up to 52 weeks post-injection in 17% of participants [13], while the HPTN 077 trial reported a 33% longer half-life in females [12].

Rilpivirine nanosuspensions maintain systemic concentrations for 3–6 months, necessitating careful management of the pharmacokinetic tail to prevent resistance [14]. Risk factors for virological failure include pre-existing rilpivirine resistance, HIV-1 subtype A6/A1, and BMI ≥ 30 kg/m^2^, particularly during the initial 8 weeks of therapy [15].

Multiple clinical trials have confirmed the efficacy and safety of CAB + RPV. The LATTE-2 trial demonstrated 94% virological suppression at week 96 with bimonthly injections [16], while the POLAR study reported 98% suppression following a switch from oral therapy [17]. The FLAIR and ATLAS trials showed 93% suppression at week 48, with minimal virological failure [18].

The SOLAR study [19] found that CAB + RPV was non-inferior to daily oral BIC/FTC/TAF, with improved patient-reported outcomes. The CARES trial in sub-Saharan Africa confirmed comparable efficacy between LA and oral therapy, though resistance mutations emerged in two cases [20].

The CARISEL study [21] demonstrated high acceptability among European healthcare providers, and the HOLA study highlighted the feasibility of administration in both hospital and community settings [22].

In light of this growing evidence base, we conducted a retrospective multicenter study involving 160 virologically suppressed PLWH across eight Italian infectious disease units. Participants received intramuscular CAB (600 mg) and RPV (900 mg) every eight weeks without an oral lead-in phase. This study aims to evaluate the real-world efficacy, safety, and tolerability of CAB + RPV, contributing to the expanding literature on LA-ART.

## 2. Materials and Methods

The cohort was initially composed of 160 participants, whose data were systematically collected at baseline (at the time of switching to CAB/RPV) and after 24 weeks of CAB + RPV administration, notably without implementing an initial oral CAB + RPV induction period. People living with HIV (PLWH) received an initial cabotegravir intramuscular loading dose of 600 mg. The same dosage was used for the loading dose administered after four weeks and the maintenance injections administered every eight weeks. The same administration scheme was followed for Rilpivirine, with a dosage of 900 mg. The analytical framework included exclusively those PLWH who completed a minimum of four injection cycles, representing 24 weeks of continuous treatment at the Infectious Diseases Units of ARNAS “Garibaldi Nesima” Hospital, University of Catania, Department of Clinical and Experimental Medicine, Catania, Italy; the Unit of Infectious Diseases, “G. Martino” University Hospital, University of Messina, Department of Clinical and Experimental Medicine, Messina, Italy; the Unit of Infectious Diseases, Department of Medicine, Surgery and Pharmacy, University of Sassari, Sassari, Italy; the Unit of Infectious Diseases, Department of Medical and Surgical Sciences, University of Foggia, 71122 Foggia, Italy; the Infectious Diseases Unit Umberto I hospital, ASP Siracusa, Italy; the Unit of Infectious Diseases, “G. Rodolico—S. Marco” University Hospital, Catania, Italy; the Unit of Infectious Diseases, AOE “Cannizzaro”, Catania, Italy; the ASP Ragusa, Infectious Diseases, Ragusa, Italy. Patients were included in the study only after verification of compliance with the inclusion criteria, which included: at least six months of adherence to antiretroviral therapy; absence of mutations associated with resistance to cabotegravir or rilpivirine, neither documented nor suspected; and above 18 years of age. Pregnant women were excluded from the study, even though all other parameters complied with the inclusion criteria.

Clinical and biochemical parameters were recorded within the Shine-Shic database and considered. Biochemical and virological data such as: CD4+ and CD8+ T Lymphocytes absolute count; CD4+/CD8+ Ratio; HIV-RNA plasmatic viral load (VL); serum creatinine levels; total cholesterol levels; HDL cholesterol levels; triglycerides levels were analyzed by each single HIV clinic in laboratories accredited according to ISO/IEC 17025, and using instruments bearing the CE-IVD marking in compliance with Regulation (EU) 2017/746.

To quantify VL, we used a quantitative RT-PCR with a detection limit of 20 HIV copies/mL. The measurements with undetectable viremia were recorded as TND (target not detected). Virological failure (VF) and blips were recorded. VF was defined as >200 HIV copies/mL in 2 consecutive measurements or a single VL > 1000 copies/mL. While blips were defined as 50–1000 HIV copies/mL in a single detection, preceded and followed by undetectable viremia. Finally, very low-level viremia was defined as persistent detection of HIV–RNA ranging from 20 to 50 copies/mL.

Adverse events were actively monitored on the day of drug administration through both clinical examination and standardized questionnaires. Additionally, patient self-reported symptoms were systematically recorded. To record the adverse site reactions, we referred to the Division of AIDS (DAIDS) Table for grading the severity of adult and pediatric adverse events [23].

Clinical data distribution was analyzed using the Shapiro–Wilk statistical test to choose the appropriate statistical tests for comparisons between timepoints. Paired *t*-test was used to analyze normally distributed variables; non-normally distributed variables were analyzed using the Wilcoxon matched pairs signed-rank test.

Data collection, processing, and documentation were systematically performed using Microsoft Excel spreadsheet applications to facilitate appropriate 24-week comparative analyses. All the statistical analyses were performed using GraphPad Prism version 9.5.1 for macOS, GraphPad Software, Boston, MA, USA, www.graphpad.com.

## 3. Results

The study population consisted of 72.4% male participants, with a median age of 49 years (IQR 37–61). Among the 160 participants, data on the duration of continuous virological suppression prior to switching to long-acting CAB/RPV were available for only 54 individuals (see Table 1). In this subgroup, the median duration of suppression was 7 years (IQR: 4–11).

Cohort descriptive statistics are listed in Table 1.

Treatment discontinuation occurred in six patients due to the following reasons: anxiety/depression (n = 2), adverse events including headache (n = 1), injection site reactions (n = 2), being one of them an excessive injection-associated pain and the other one swelling and erythema 2 days after the injection, and virologic failure (n = 1).

During the study, 6 PLWH out of 160 reverted to their previous oral regimen, but they remained virologically suppressed. Parameters affected by significant variations are described in Table 2 using mean ± standard deviation (SD) or median ± interquartile range (IQR), respectively, for normally distributed variables and for non-normally distributed ones. At the 24-week (24 w) follow-up, 154 out of the 160 PLWH who reached this timepoint, corresponding to 96.25%, maintained virological suppression (VS), while the proportion of individuals with HIV RNA classified as target not detected (TND) increased from 71% (114) at baseline to 76% (121) at week 24. Only one case of virological failure was recorded (>200 copies/mL in 2 consecutive measurements), and among the two participants who presented with a viral load greater than 50 copies/mL at baseline, both achieved virological suppression by week 24. Furthermore, five individuals with persistent very low-level viremia (19–50 copies/mL) before their switch to CAB/RPV, experienced a transient increase in viral load, with values between 50 and 200 copies/mL, commonly referred to as a “blip”.

Table 3 represents the individual viral load (VL) trends from Baseline (last measured VL before switching to CAB/RPV) to the endpoint (24 weeks after switching to LA CAB/RPV).

**Figure 1:**
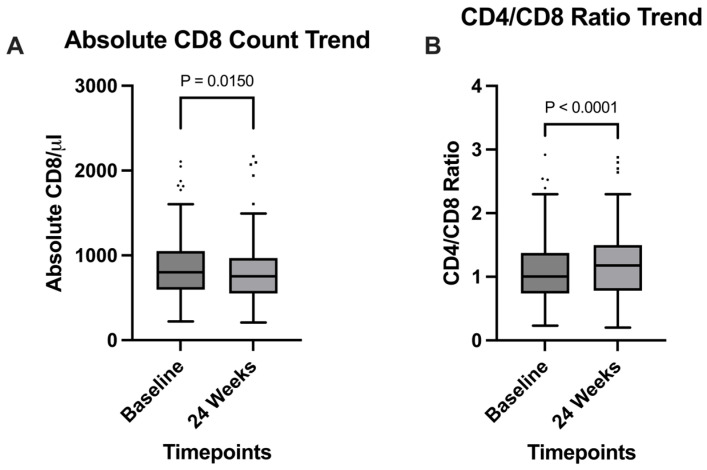
Comparison of (**A**) Absolute CD8 cell count and (**B**) CD4/CD8 ratio between the timepoints. A significant increase in the CD4/CD8 ratio was observed from the start of the study to the 24 weeks timepoint. A significant decrease in absolute CD8+ T cells per microliter was also evidenced.

**Figure 2:**
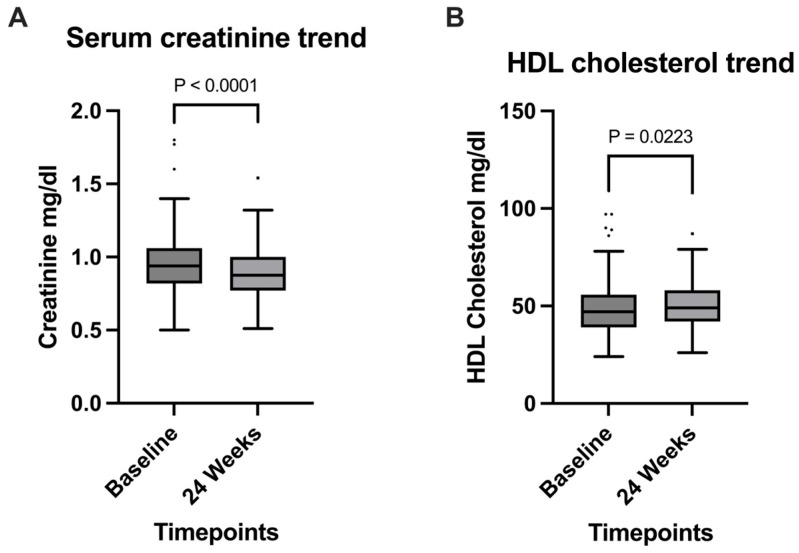
Comparison of (**A**) Serum creatinine levels and (**B**) HDL cholesterol levels between timepoints. Creatinine levels significantly decreased from the start of the study to the 24-week timepoint; A significant increase was observed for HDL cholesterol levels.

Among the clinical parameters analyzed, a significant increase in CD4+/CD8+ ratio was observed (*p* = 0.0038), going from 1 (0.74–1.4) at baseline, to 1.2 (0.78–1.5) at the 24 weeks timepoint (Figure 1B); this observation was supported by the significant decrease in absolute CD8+ T cells count, expressed in cells/mm^3^, which went from 801 (597–1051) at baseline to 754 (551–969) after 24 weeks (*p* = 0.015) (Figure 1A). Serum creatinine also significantly decreased (*p* < 0.0001) from 0.94 mg/dL (0.5–1.1) to 0.88 mg/dL (0.77–1) during the 24 weeks timespan (Figure 2A); HDL cholesterol levels showed a significant increase from a median of 47 mg/dL (39–56) at baseline to 49 mg/dL (42–58) at 24 weeks (*p* = 0.0223), reflecting potential cardiovascular benefits (Figure 2B).

## 4. Discussion

At the 24-week follow-up, 96.25% of participants maintained virological suppression, with the proportion of individuals classified as target not detected (TND) increasing from 71% at baseline to 76%. These findings align with previous clinical trials of long-acting cabotegravir and rilpivirine (CAB/RPV), which demonstrated robust virological efficacy [18,24].

Despite the increase in the proportion of TND patients, the overall percentage of patients below 50 copies/mL decreased from 98% at baseline to 96% at the 24-week timepoint. We chose to focus on the proportion of patients with TND (Target Not Detected), as recent literature suggests that achieving TND is statistically associated with a lower risk of therapeutic failure compared to low-level viremia [25] and is also linked to reduced systemic inflammation. Notably, the four patients who experienced an increase in viremia from TND to <50 copies/mL subsequently returned to the TND range during follow-up visits, indicating minimal and transient fluctuation.

Only one case of virological failure (VF) was observed (0.6%), and both participants with baseline HIV-RNA > 50 copies/mL achieved suppression by week 24. Compared to the ATLAS and FLAIR trials, which reported VF rates of 1.2% [18], our cohort exhibited a lower incidence, possibly attributable to differences in sample size and study design.

Our results are consistent with real-world data, such as the Swiss HIV cohort, which similarly reported high rates of virological suppression [26].

Five participants experienced transient viral load increases (“blips”) ranging from 50 to 200 copies/mL, consistent with the Delphi consensus definition [27], and comparable to the 7% blip rate reported in the Swiss cohort [26].

While our study focused on virologically suppressed individuals, it is important to note that those with adherence challenges, who may benefit most from LA-ART, were excluded. Evidence from Ward 86 HIV Clinic in the U.S. supports the utility of LA-ART in this population, with 54 of 57 participants achieving suppression despite prior detectable viremia [28].

Immunologically, we observed a significant increase in the CD4+/CD8+ ratio (*p* < 0.0001), rising from a mean of 1.0 at baseline to 1.2 at week 24. This improvement is consistent with prior studies [29] and was accompanied by a significant reduction in CD8+ T-cell count (*p* = 0.0150), suggesting decreased immune activation [30].

Upon stratification by prior antiretroviral regimen, statistically significant changes in CD4/CD8 ratio were observed exclusively among participants previously receiving bictegravir-based regimens (see Supplementary Table S1).

Similar trends were reported in the Milan and Padova cohorts [31], which reported an increase in the CD4+/CD8+ ratio, and in the Padova cohort [32], reinforcing the relevance of immunological recovery in mitigating chronic inflammation, a persistent challenge in PLWH despite virological control. Chronic immune activation contributes to elevated risks of cardiovascular disease, neurocognitive impairment, and non-HIV-related malignancies [33,34,35,36].

Renal safety is a key concern in ART, particularly with tenofovir-based regimens. In our study, CAB/RPV was associated with a statistically significant decrease in serum creatinine (*p* = 0.0001), suggesting improved renal function.

Upon stratification by prior antiretroviral regimen, statistically significant changes in serum creatinine levels were observed exclusively among participants previously receiving dolutegravir and bictegravir-based regimens (see Supplementary Table S1).

Cabotegravir is primarily metabolized hepatically and does not affect renal function, while rilpivirine may inhibit OCT2 transporters, potentially increasing creatinine levels [10]. However, no such increase was observed in our cohort. These findings are supported by case reports and small studies showing stable renal function following a switch to LA CAB/RPV, even in patients with pre-existing renal impairment [37,38,39].

We also explored the impact on lipid profile: in our study, we observed a statistically significant increase in HDL cholesterol at 24 weeks (*p* = 0.0223), reflecting potential cardiovascular benefits.

Upon stratification by prior antiretroviral regimen, statistically significant changes in HDL cholesterol were observed exclusively among participants previously receiving dolutegravir-based regimens (see Supplementary Table S1).

An increase in HDL in PLWH on LA CAB/RPV has been reported in other studies as well, even though it was not always statistically significant [30,40].

Pain at the injection site was the primary side effect reported in our study, mirroring results from the ATLAS and FLAIR trials, where injection site reactions (ISRs) were common but generally mild to moderate in severity [18]. Nonetheless, participants described LA-ART as convenient, discreet, and less stigmatizing than daily oral therapy, with reduced risk of inadvertent HIV status disclosure.

Overall, our findings confirm the efficacy, safety, and tolerability of CAB/RPV in a real-world setting. This study contributes valuable data from a relatively large cohort, supporting the integration of LA-ART into routine clinical practice and highlighting its potential to improve outcomes in diverse populations.

Given that our principal findings concern metabolic and immunological changes, and that the baseline consisted of a group of stable, virosuppressed PLWH prior to switching to CAB/RPV, our results are limited by the relatively short follow-up period (24 weeks). Extended observation is warranted to further assess the efficacy and long-term effects of LA CAB/RPV. A limitation of our study is also the limited sample size, which did not allow for adequately powered stratified analyses by both prior antiretroviral regimen and comorbid conditions. As such, any subgroup comparisons would risk yielding unreliable estimates. Future studies with larger cohorts are warranted to explore these associations in greater detail and to validate our findings across more diverse clinical contexts. Potential biases may arise from the retrospective observational design of the study. An additional limitation is the heterogeneity of baseline ART regimens prior to the switch to LA CAB/RPV.

## 5. Conclusions

Before regulatory approval of the first long-acting injectable therapy, patient surveys demonstrated substantial interest in injectable formulations, mostly because, for certain individuals, daily oral therapy represented an unwelcome constant reminder of their HIV diagnosis.

Long-acting efficacy and safety have been extensively evaluated in both controlled trials and real-world clinical settings. Pivotal studies, including ATLAS and FLAIR, demonstrated non-inferiority to daily oral therapy and sustained suppression in patients who were virologically suppressed at initial injection.

At week 24, statistical analysis demonstrates that long-acting cabotegravir + rilpivirine use in real-world settings not only maintains virological suppression but also induces a reduction in the CD8+ T-cell population and increases the CD4+/CD8+ ratio. Those immunological changes could indicate diminished inflammatory activity that, if confirmed by larger cohorts and longer follow-up, could be an important strength of the LA CAB/RPV regimen. Additionally, our study’s observation of decreased creatinine levels and elevated HDL concentrations is an important indicator of the long-term safety and sustainability of this antiretroviral regimen.

Long-acting therapy represents an important tool for those struggling with adherence due to social, structural, and behavioral barriers; understanding the impact of this treatment in these groups is essential. Additional data are needed to assess safety in pregnant women, for whom nausea, vomiting, and morning sickness compromise oral therapy adherence, and in adolescents who characteristically struggle to achieve high adherence rates.

## Figures and Tables

**Table 1:** Participants Characteristics.

Characteristic	Value
Number of PLWH	160
Median Age	49 (IQR 37–61)
Patients who did not perform the switch	6 (3.75%)
Male patients	115 (72.4%)
Time (years) from infection, median (IQR)	11 (6–18)
Time (years) in ART, median (IQR)	10 (7–18)
Time (years) of continuous virological suppression before switching to CAB/RPV *, median (IQR)	7 (4–11)
Patients switching from Dolutegravir (DTG)-based 2 Drug Regimens (2DR), n (%)	85 (53.12%)
Patients switching from DTG-based 3DR, n (%)	16 (10%)
Patients switching from Doravirine-based regimens, n (%)	7 (4.38%)
Patients switching from Bictegravir-based regimens, n (%)	24 (15%)
Patients switching from Tenofovir Alafenamide-based regimens, n (%)	28 (17.5%)
Patients with NNRTI history, n (%)	109 (68.12%)
Patients with InSTIs history, n (%)	136 (85%)
Route of transmission:	
Sexual, n (%)	115 (71.9%)
IDU, n (%)	16 (10%)
Uncertain, n (%)	29 (18.1%)
Comorbidities:	
Diabetes, n (%)	9 (5.63%)
Hypertension, n (%)	37 (23.13%)
Dyslipidemia, n (%)	48 (30%)
Heart failure, n (%)	4 (2.5%)
Prostatic hypertrophy, n (%)	9 (5.63%)
Chronic Obstructive Pulmonary Disease (COPD), n (%)	19 (11.9%)
Anxiety, n (%)	18 (11.25%)
Depression, n (%)	12 (7.5%)
Steatosis, n (%)	2 (1.25%)
Cirrhosis, n (%)	3 (1.88%)

Table 1: Summary of demographic and clinical data of Participants (absolute number and percentage). * 54/160 patients analyzed

**Table 2:** Resume of parameters between baseline and endpoint of the study.

Parameter	Value. at BL	Value at 24w	*p* Value
Serum creatinine (mg/dL, median, IQR)	0.94 (0.5–1.1)	0.88 (0.77–1)	0.0001
HDL Cholesterol (mg/dL, median IQR)	47 (39–56)	49 (42–58)	0.0223
CD4/mm^3^ (Median, IQR)	830 (640–1076)	826 (661–1061)	0.8708
CD8/mm^3^ (Median, IQR)	801 (597–1051)	754 (551–969)	0.0150
CD4/CD8 Ratio (Median, IQR)	1 (0.74–1.4)	1.2 (0.78–1.5)	0.0038
Virological suppression	158	154	-

Table 2. Resume of parameters affected by statistically significant variations. Mean ± SD was used to describe normally distributed variables, while Median ± IQR was used to describe variables that did not follow a normal distribution.

**Table 3:** Viral load (VL) trends.

	TND	19–50	51–200	>200
BL	114 (71%)	44 (27%)	1 (1%)	1 (1%)
24 Weeks	121 (76%)	33 (20%)	5 (3%)	1 (1%)

## Data Availability

The data presented in this study are available upon request from the corresponding author.

## Supplementary Materials

The following supporting information can be downloaded at: https://www.mdpi.com/article/10.3390/v17111417/s1. Supplementary Table S1. Summary of parameter changes based on the most recent oral regimen before switching to LA agents.

## Abbreviations

The following abbreviations are used in this manuscript:

EMA	European Medicines Agency
FDA	Food and Drug Administration
LEN	Lenacapavir
PLWH	People living with HIV
CVF	Confirmed virologic failure
LA	Long-acting
ISR	Injection site reaction
TND	Target not detected
CAB/RPV	Cabotegravir/rilpivirine
TAF	Tenofovir alafenimide
BIC	Bictegravir
BL	Baseline
