# Supplementary material for: Efficacy and Safety of Cabotegravir–Rilpivirine in PLWH: A Real-World Study
Source: Viruses. 2025 Oct 24;17(11):1417. doi: 10.3390/v17111417 (PMC12656754; doi:10.3390/v17111417)
Supplement: Supplementary file 1 [file viruses-17-01417-s001.zip › viruses-3935506-supplementary.pdf]

Supplementary Table S1. *Resume of parameters variations according to the last oral regimen received just before the switch to LA agents. Mean ±SD was used to describe normally distributed variables, while Median ±IQR was used to describe variables that didn't follow a normal distribution. Statistically significant changes are shown in bold characters.*

Regimen	Parameter	Baseline	24 Weeks	p-value
**DTG 2DR**	Total cholesterol, mg/ml, Mean (±SD)	186 (± 39)	182 (±45)	0,8878
	HDL cholesterol, mg/ml, Mean (±SD)	50 (± 16)	53 (±14)	**0,0112**
	CD4/CD8 ratio, Mean (±SD)	1,1 (± 0,50)	1,2 (± 0,48)	0,0953
	Serum creatinine, mg/ml, Mean (±SD)	0,99 (± 0,19)	0,90 (± 0,20)	**<0,0001**
**DTG 3DR**	Total cholesterol, mg/ml, Mean (±SD)	170 (± 19)	183 (±30)	0,0746
	HDL cholesterol, mg/ml, Mean (±SD)	45 (±10)	55 (±11)	**0,0141**
	CD4/CD8 ratio, Mean (±SD)	1,3 (± 0,58)	1,3 (± 0,56)	0,4134
	Serum creatinine, mg/ml, Mean (±SD)	0,95 (± 0,14)	0,86 (± 0,16)	0,0792
**Doravirine-based**	Total cholesterol, mg/ml, Mean (±SD)	143 (± 20)	153 (± 39)	0,5332
	HDL cholesterol, mg/ml, Mean (±SD)	44 (± 11)	50 (± 16)	0,3805
	CD4/CD8 ratio, Mean (±SD)	1,1 (± 0,68)	1,3 (± 0,47)	0,3836
	Serum creatinine, mg/ml, Mean (±SD)	0,90 (± 0,07)	0,84 (± 0,11)	0,1865
**Bictegravir-based**	Total cholesterol, mg/ml, Mean (±SD)	196 (± 32)	196 (± 32)	0,7079
	HDL cholesterol, mg/ml, Mean (±SD)	49 (± 10)	49 (± 15)	0,9045
	CD4/CD8 ratio, Mean (±SD)	1,1 (± 0,55)	1,2 (± 0,53)	**0,0227**
	Serum creatinine, mg/ml, Mean (±SD)	0,99 (±0,26)	0,86 (±0,18)	**0,0265**
**TAF-based**	Total cholesterol, mg/ml, Mean (±SD)	182 (± 40)	189 (± 45)	0,5027
	HDL cholesterol, mg/ml, Mean (±SD)	50 (± 15)	50 (± 12)	0,8781
	CD4/CD8 ratio, Mean (±SD)	1,1 (± 0,46)	1,1 (± 0,51)	0,8610
	Serum creatinine, mg/ml, Mean (±SD)	0,90 (± 0,16)	0,95 (± 0,16)	0,2814
